# TREND database: Retinal images of healthy young subjects visualized by a portable digital non-mydriatic fundus camera

**DOI:** 10.1371/journal.pone.0254918

**Published:** 2021-07-23

**Authors:** Natasa Popovic, Stela Vujosevic, Miroslav Radunović, Miodrag Radunović, Tomo Popovic

**Affiliations:** 1 Faculty of Medicine, University of Montenegro, Podgorica, Montenegro; 2 Eye Clinic, IRCCS MultiMedica, Milan, Italy; 3 Faculty for Information Systems and Technologies, University of Donja Gorica, Podgorica, Montenegro; Nicolaus Copernicus University, POLAND

## Abstract

**T**opological characterization of the **R**etinal microvascular n**E**twork visualized by portable fu**ND**us camera (**TREND**) is a database comprising of 72 color digital retinal images collected from the students of the Faculty of Medicine at the University of Montenegro, in the period from February 18^th^ to March 11^th^ 2020. The database also includes binarized images of manually segmented microvascular networks associated with each raw image. The participant demographic characteristics, health status, and social habits information such as age, sex, body mass index, smoking history, alcohol use, as well as previous medical history was collected. As proof of the concept, a smaller set of 10 color digital fundus images from healthy older participants is also included. Comparison of the microvascular parameters of these two sets of images demonstrate that digital fundus images recorded with a hand-held portable camera are able to capture the changes in patterns of microvascular network associated with aging. The raw images from the TREND database provide a standard that defines normal retinal anatomy and microvascular network geometry in young healthy people in Montenegro as it is seen with the digital hand-held portable non-mydriatic MiiS HORUS Scope DEC 200.This knowledge could facilitate the application of this technology at the primary level of health care for large scale telematic screening for complications of chronic diseases, such as hypertensive and diabetic retinopathy. In addition, it could aid in the development of new methods for early detection of age-related changes in the retina, systemic chronic diseases, as well as eye-specific diseases. The associated manually segmented images of the microvascular networks provide the standard that can be used for development of automatic software for image quality assessment, segmentation of microvascular network, and for computer-aided detection of pathological changes in retina. The TREND database is freely available at https://doi.org/10.5281/zenodo.4521043.

## Introduction

Examination of retinal fundus images is a well known and widely accepted diagnostic approach used to diagnose numerous ocular diseases, as well as to detect ocular complications of some chronic systemic diseases. More recently, retinal microvascular network patterns have also been studied extensively for their use in early detection of ocular and systemic diseases, as well as physiologic changes associated with normal aging [1, 2]. For example, decreased retinal microvascular complexity has been found in hypertension and diabetes [3–6]. Certain regional patterns of decreased retinal vascular complexity measured by multifractal and lacunarity analysis have been found to correlate with cognitive impairment in the aging population [7]. In addition, decreased vascular complexity measured by fractal analysis is observed in glaucoma [2]. For this reason, the methods used to characterize retinal microvascular network geometry currently yield relatively non-specific information. From this perspective, the method of visualization of the retinal microvasculature through the eye could be currently considered as “a window to health”, because it allows the assessment of general health status of an individual by applying non-invasive technologies. The specificity of this approach could be increased in the future by characterization of microvascular patterns associated with certain conditions by using more than one parameter at a time [8]. These research efforts will require studying large data sets. In that regard, development of efficient and accurate computer-aided image analysis systems to study these data sets could save time [9].

The studies examining the eye fundus most frequently rely on the use of sophisticated equipment that is usually available exclusively in specialized health care institutions. However, thanks to numerous advances in technology in the course of the past several years, portable hand-held fundus cameras are becoming more affordable and the images captured by using this technology are improving in quality. In addition, the use of a portable fundus camera has numerous advantages, especially when used in the primary health care setting. For example, they are lightweight, do not require long training, and are easy to use in any setting including on the patients with limited mobility. These cameras do not require a dilated pupil in order to capture a good quality digital image of the eye fundus [10].

In order for this retinal imaging technology to be applied successfully in practice, it is necessary that there is first a detailed description of the healthy retinal microvascular network geometry visualized through this method. Therefore, the goal of our study was to describe the morphology of the retinal microvascular network in young and healthy subjects as it appears when it is captured by a portable digital fundus camera. These findings will represent the foundation and reference point when using the same approach to detect microvascular changes associated with disease, or with the normal physiology of aging. They will also represent the standard that can be used for development of automatic software for image quality assessment, segmentation of microvascular network, and for computer-aided detection of pathological changes in retina.

There are published databases of retinal fundus images captured with a same type of hand-held digital camera, but these images are either proprietary [9], or they may be available on a reasonable request [10]. There are other freely available databases of retinal fundus images like STARE [11], DRIVE [12], DIARETDB1 [13], HRF [14, 15], DRIMDB [16, 17]. However, some of them are comprised of a large number of low resolution and low quality images, and a very small subset of images is associated with manually segmented microvascular networks (STARE, DRIVE). In other cases, the high quality images are captured with advanced equipment that is usually not readily available in the primary health care environment (Messidor [18], HRF). Moreover, unlike the database we present in this paper, these databases usually contain images obtained from the patients with a specific health condition, and a very small subset of images comes from healthy individuals (Messidor, DIARETDB1, HRF, DRIMDB), Table 1.

**Table 1:** Other similar retinal image databases and their availability.

Database name and retinal pathology	FOV*	Resolution	Type of camera	Number of color images	Number of manually segmented images	Availability
**STARE Various**	35°	700 X 605	TopCon TRV-50	402	20, 37 [19]	Freely available [11]
**DRIVE Diabetic retinopathy**	45°	584 X 768	Canon CR5 non-mydriatic 3CCD	40	40	Freely available [12]
**Messidor Diabetic retinopathy**	45°	1444 X 960 2240 X 1488 2304 X 1536	3CCD camera on a Topcon TRC NW6 non-mydriatic retinograph	1200	None	On a reasonable request [18]
**HRF Diabetic retinopathy, glaucoma**	45°	3504 X 2336	Canon CR-1	45	45	Freely available [14, 15]
**DIARETDB1 Diabetic retinopathy**	50°	1500 X 1152	ZEISS FF 450^plus^ fundus camera with Nikon F5 digital camera	89	None	Freely available [13]
**DRIMDB Diabetic retinopathy**	60°	570 X 760	Canon CF-60UVi	216	None	Freely available [16, 17]
**LOCAL1, Not specified**	60°	2500 X 1960	MiiS HORUS Scope DEC 200	199	None	Proprietary [9]
**LOCAL2, Not specified**	60°	2500 X 1960	MiiS HORUS Scope DEC 200	103	None	Proprietary [9]
**Jin et al. 2017 Various**	60°	8 Megapixels	MiiS HORUS Scope DEC 200	400	None	On a reasonable request [10]
**TREND Healthy**	45°	2560 X 1920	MiiS HORUS Scope DEC 200	72	72	Freely available

* Field of view (FOV)

In the present study, we analyzed the microvascular networks on images of 72 eyes, including both eyes of 32 young healthy subjects, and one eye of 8 young healthy subjects. The images were captured by using portable hand-held camera, MiiS HORUS Scope DEC 200. To describe the healthy young population, the information on the following constant variables was collected: sex, age, body mass index, alcohol use, and smoking status. The vascular trees in each image were manually segmented, and the geometry of the skeletonized microvascular network was described by measuring the box-counting fractal dimension, lacunarity dimension, multifractal dimensions, vessel percentage area, total number of vessel junctions, and the total vessel length.

In addition, the quality of images used in the study was assessed in three ways: visually by an ophthalmologist; by performing descriptive statistical analysis; and by comparing the parameters of the microvascular networks between the left and right eye of the same person, in cases where images from both eyes were available.

Finally, the utility of this database was demonstrated. As a proof of the concept, a smaller set of 10 color digital fundus images from healthy older participants was also included. The 82 retinal images were used to show that differences in age, gender, body mass index (BMI), alcohol, and tobacco use among the participants translate to changes in retinal microvascular patterns, and that the observed changes are similar to those already described by others [1, 20–22].

The TREND database is freely available at https://doi.org/10.5281/zenodo.4521043 for other researchers to use.

## Materials and methods

### Ethical approval and informed consent

The TREND study was approved by the Ethical Committee of the Faculty of Medicine of the University of Montenegro (Protocol No. 2487/4). A total of 43 participants were recruited among the medical and dental school students between February 18^th^ and March 11^th^ 2020.

In addition to this, the eye fundus images from 10 healthy older participants have been collected in the period from December 22^th^ 2020. to January 21^th^ of 2021. The data collection from this group was approved by the Ethical Committee of the Faculty of Medicine of the University of Montenegro and the Ethical Committee of Clinical Center of Montenegro (Protocol No. 3824/4, and Protocol No. 03/01-11417/1).

### Inclusion and exclusion criteria

For the healthy young group (TREND database), the subjects qualified to participate in the study if they were between 18 and 25 years of age and had no history of any chronic systemic disease requiring medical management. The main exclusion criteria were the presence of chronic ocular pathology like high myopia greater than -5 diopters (D), opacity of clear eye media preventing good quality imaging of the fundus, and pathologic changes of retina interfering with the segmentation of retinal microvasculature in the affected area.

For the healthy, older subjects, the following inclusion criteria were used: age 55 or older, negative history of cancer, negative history of poorly controlled systemic illness (such as diabetes mellitus, coronary artery disease, cerebrovascular disease, or hypertension), no history of stroke, and no dementia or other neurological disease. Exclusion criteria were current acute illness, current drug or alcohol abuse, or uncompensated psychiatric disease.

### Participant characteristics

After signing the informed consent, all participants filled a short standardized questionnaire, which included questions pertaining to the following variables: age, sex, weight, height, previous medical history, concomitant medications, and social history (potential use of alcohol, tobacco, and drugs). Alcohol users were defined as those participants who drink any amount of alcoholic beverages daily or occasionally, while alcohol non-users stated they never drink alcohol. Current smokers were defined as participants who smoke any number of cigarettes daily or on some days, while non-smokers were those who never smoked or are past smokers. Weight and height were used to calculate the BMI according to the formula BMI = weight(kg)/height^2^(m^2^). Participants with a BMI between 18.5 and 24.9 kg/m^2^ were considered to have normal (healthy) weight, those with BMI between 25 and 29.9 kg/m^2^ overweight, and those over 30 kg/m^2^ were considered obese. Previous ocular history including refraction error was collected as well.

### Fundus images

Systemic circulation has a tree-like structure in which the largest blood vessels originate from the heart. The diameters of blood vessels gradually decrease with subsequent branching. The arterial vascular tree carries oxygenated blood to peripheral tissues, while the venous tree carries deoxygenated blood in the opposite direction. The smallest branches of the arterial tree that belong to the highest order of branching are called arterioles. Similarly, the smallest branches of the venous tree are called venules. Arterioles and venules are connected through capillaries, which are the blood vessels with the smallest diameter. The primary function of capillaries is nutrient and gas exchange between the blood and the surrounding tissue.

The largest blood vessels in retinal images originate in the optic disk area and belong to the category of small arteries and small veins. They further branch giving rise to the arterioles and venules, so the blood vessels seen at 0.5 diameters from the optic disc margin are arterioles and venules. While microvascular network consists of arterioles, capillaries and venules [23], capillaries are not normally visualized by the retinal fundus camera. The portable fundus camera used in the present study can capture blood vessels up to the 3^rd^ generation of branching, which also belong to the category of arterioles and venules [2, 7].

The fundus images in the present study were acquired by using the non-mydriatic hand-held portable MiiS HORUS Scope DEC 200, manufactured by Medimaging Integrated Solutions Inc from Taiwan. The images were captured with a 45° FOV and have 2560 X 1920 pixel resolution. The automatic focus and capture settings of the camera were used with FOV centered between the macula and the optic disc. For each image, the cameras “Aiming Light /Capture Light “was set to “IR/White LED” = 3/7. After a brief online training, a family medicine physician captured all the images. Both eyes were imaged in every participant.

### Image quality assessment by an ophthalmologist

The quality of images as well as the presence or absence of an eye fundus pathology was verified by an ophthalmologist. There are several studies delineating criteria for selection of good quality retinal images, although currently there is no widely accepted standard scale used for this purpose as these criteria could be improved to make evaluation more objective [9, 10, 17, 24]. Most frequently these criteria are developed for the purpose of screening for diabetic retinopathy [17, 24]. Since the earliest changes in diabetic retinopathy are microvascular aneurysms, the goal of the image quality assessment is to ensure that small macular vessels with similar color and dimensions as microvascular aneurysms are clearly visible. In accordance with this, the most commonly used requirements can be divided into 3 groups. The first group consists of the structural requirements which state that the 3^rd^ generation blood vessels around macula should be visible. According to the second group, the global feature requirements, the image has to be free of distortion of illumination or distortion of color, and also free of blur and poor contrast. Finally, the field definition requirement states that the fovea should be at least 2 optic disc diameters from the edge of the image, and optic disc and superior and inferior temporal vessel arcade should be visible.

**Fig 1:**
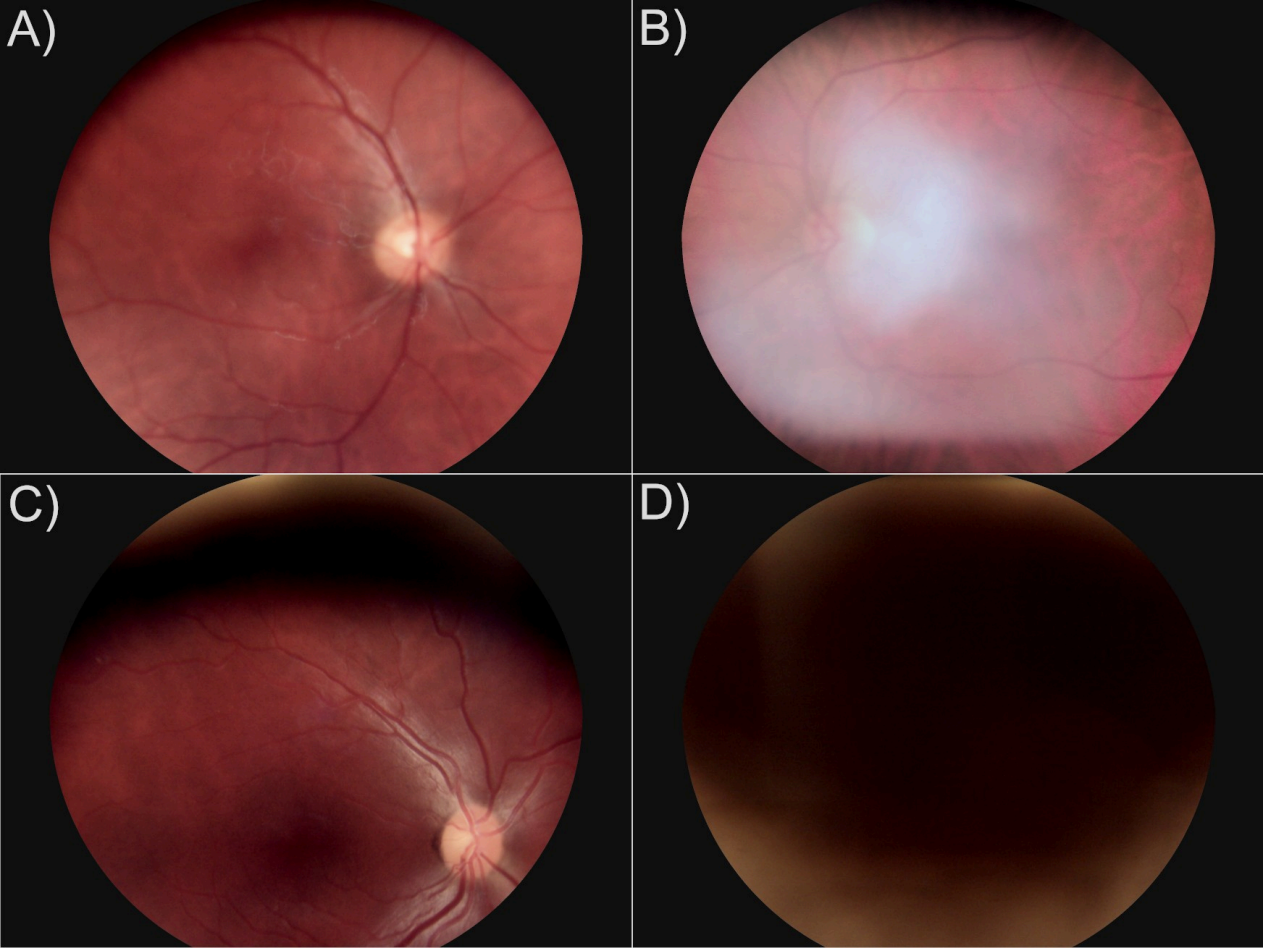
Images of inadequate quality. A) Structural feature based quality requirements not met- 3^rd^ generation blood vessels around macula are not visible; B) Global feature requirements are not met- illumination distortion; C) Field definition and global feature requirements are not met- more than 25% of the image has color distortion, the fovea is less than 2 optic disc diameters from the edge, and most of the lower temporal vessel arcade is not visible. D) No retinal features can be recognized, the patient blinked.

The present study focuses on the spatial features of the microvascular network. The region between 0.5 to 2 optic disc diameters has been accepted as the standard area of interest for this type of measurements [1, 25, 26]. Therefore, the above mentioned three groups of criteria were slightly modified, so the presence of illumination or color distortion in up to 25% of the image at the periphery was tolerated. Also, if less than 50% of the one temporal arcade is not included in the image, the image would still be considered as of good quality. Examples of the images of inadequate quality are shown in the Fig 1.

### Manual segmentation

The manual segmentation of the color fundus images that were determined to be of satisfactory quality was performed by a family medicine doctor, and verified by an ophthalmologist. The segmentation was accomplished using the Vampire software to yield the binarized 8-bit images of the retinal microvascular network in png format [27].

### Box-counting, lacunarity, and multifractal analyses

The digital fundus images were skeletonized in ImageJ 1.53a software version [28] by using Skeletonize 2D/3D plugin. To define scaling features used to sample the image, the box-counting dimension (Db) and lacunarity dimension (Λ) of the skeletonized images were calculated by using FracLac plugin with the minimum box size set to 0 pixels, and the maximum box size to 45% of the image. To define the scaling features in the multifractal analysis, the minimum box size was set to 10 pixels and maximum box size to 60%. Multifractal analysis was used to determine the capacity dimension (D_0_), information dimension (D_1_) and correlation dimension (D_2_) as previously explained [4].

### Angiotool analysis

The vessel percentage area, total number of junctions, and total vessel length were measured on the skeletonized images using the Angiotool software that was downloaded separately [29, 30].

### Statistical analysis

Descriptive statistics, paired sample and independent sample t-tests, 2-way analysis of variance (ANOVA), and Pearson’s correlation were performed in the statistical analysis software R. P-values <0.05 were considered statistically significant.

## Results and discussion

### Formation of the image database

In most of the cases, 1 imaging attempt per each eye was sufficient to capture a good quality image. Not more than 3 attempts per eye were made. In young healthy participants the imaging success rate, measured as a percent of participants with one good quality image obtained for at least one eye, was about 90%. These results are comparable to the results published by other researchers using the same or similar type of camera. For example Jin et al. report 83.5% fundus photographs captured by using Miis HORUS Scope DEC 200 had excellent and good quality [10]. Yogesan et al. report ed that 93% of photographs captured by Nidek hand-held camera were of good quality [31].

In older patients, the imaging success rate was about 70%. Difficulties encountered during the imaging were motion artifacts, media opacities, and small pupil size that was not pharmacologically dilated. Jin’s group evaluated the image quality of 400 images captured with the same type of camera in subjects whose age ranged from 9 to 84 years. They also reported media opacities, pupil size, and fundus reflection as the main factors affecting the quality of the image [10].

Out of 43 healthy young subjects included in the TREND study, the data collected from 3 subjects were excluded from the final data set because of the low quality images from both eyes. In 7 cases, the image quality was satisfactory only in one eye, and those images were included in the final data set. In 1 participant, one eye had a choroidal nevus, so only the image from non-affected eye was included in the data set. Finally, 32 subjects had good quality images from both eyes, and 8 from one eye. Out of 10 recruited healthy older patients, good quality images were collected from both eyes from 3 participants, and the image quality was satisfactory from one eye from 4 participants, Table 2.

**Table 2:** Formation of the image database.

Number of young and healthy subjects (TREND)	Number of healthy older subjects*	Outcome
32	3	Good quality of images from both eyes
8	4	Good quality of images from one eye
3	3	Poor quality of images from both eyes
**Total: 43 subjects 72 good quality images**	**Total: 10 subjects 10 good quality images**	

* Additional set of images used to demonstrate the TREND database utility

### Technical validation

**Fig 2:**
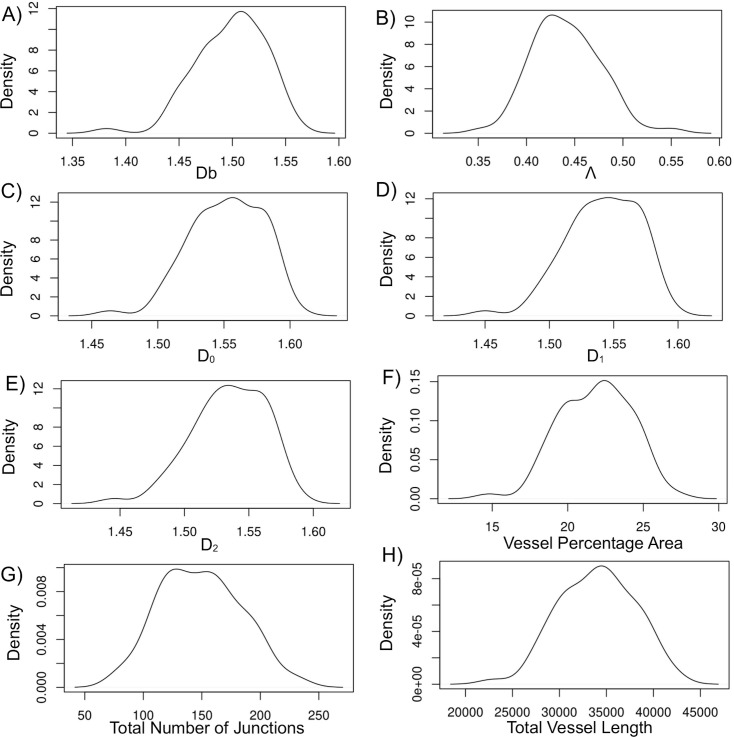
Dependent variable data distribution analysis. Data originates from all 72 fundus images of the healthy young population: A) Box-counting dimension Db; B) Lacunarity dimension Λ; C) Capacity dimension D_0_; D) Information dimension D_1_, E) Correlation dimension D_2_; F) Vessel percentage area; G) Total number of junctions; H) Total vessel length. All dependent variables have nearly normal distribution without clustering or extreme skewing.

**Fig 3:**
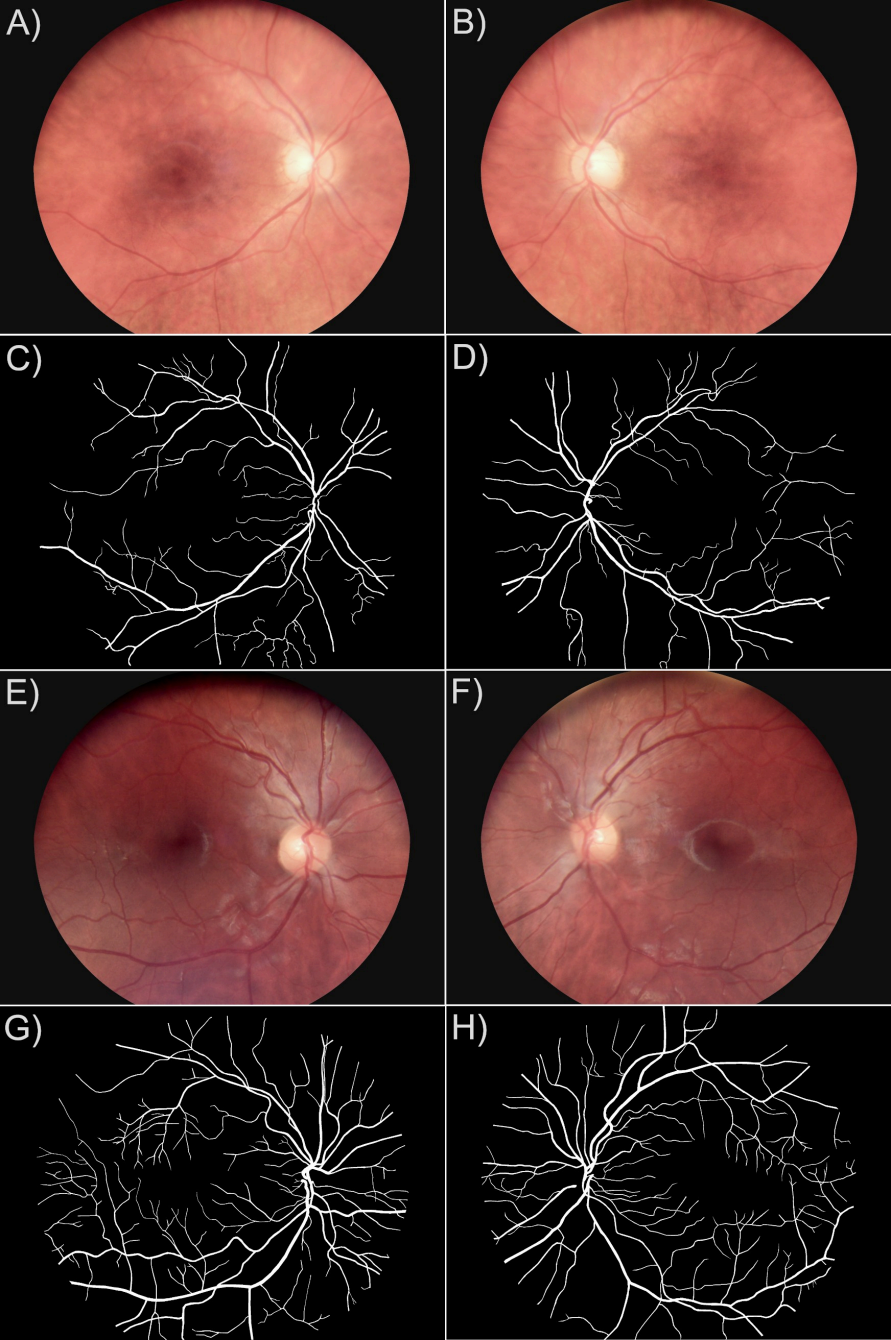
Visual comparison of images in the TREND database from the right and the left eye of the same healthy person show interocular symmetry. A) and B) digital color fundus images of the right and the left fellow eyes with low vessel percentage area; E) and F) digital color fundus images of the right and the left fellow eyes with high vessel percentage area; C), D), G) and H) are corresponding manually segmented images that display interocular symmetry as well.

The technical validation was performed in 3 ways: 1) visual inspection by an ophthalmologist to exclude low quality images (Fig 1), as described in Materials and methods section; 2) statistical analysis of the data distribution to show that all dependent variables have a close to normal distribution without extreme skewing or clustering into groups, as shown in the Fig 2; 3) comparison of the left and right eye images, both visually and by statistical analysis.

There is an increasing body of research data showing that microvascular changes in the eye reflect changes in microvascular networks in the rest of the body [1, 3–7]. In most of these studies data from only one eye is used to extrapolate these conclusions. In addition, in some cases of eye diseases when the information on the normal retinal vascular density cannot be obtained from the affected eye due to abnormal eye anatomy, this information is extrapolated from the measurements on the fellow eye that is not affected by the disease [32]. Based on these data, we assumed that there is microvascular network symmetry between the fellow eyes in healthy young people, and we used this assumption to check the quality of the TREND data set. To our best knowledge the TREND database is the first database of this kind that uses fellow eyes as control for quality of retinal images.

In accordance with this assumption, the visual inspection of the color digital fundus images and the images with segmented microvascular networks originating from the left and right eye of the same person in the TREND database showed high symmetrical relationship (Fig 3).

In order to confirm this correlation between the right and left eye objectively, we used the dependent variables such as box-counting dimension, lacunarity, multifractal dimensions (D_0_, D_1_ and D_2_), as well as vessel percentage area, total number of junctions, and total vessel length and performed the Pearson’s correlation test. Pearson’s R statistics showed moderate, but statistically significant correlation for all of these variables except for the lacunarity. At the same time, as expected, the paired sample t-test comparing images from the left and the right eyes of the same person was not able to detect any significant differences for any of these variables, Table 3.

**Table 3:** Comparison of the dependent variables between the left and the right eye.

Dependent variable being compared between left and right eye	Pearson’s correlation p-value	Pearson’s correlation R statistics	Paired sample t-test p-value
**Box-counting dimension Db**	<0.001*	0.578	0.971
**Lacunarity dimension Λ**	0.261	0.205	0.371
**Capacity dimension D**_**0**_	0.005*	0.481	0.885
**Information dimension D**_**1**_	0.001*	0.546	0.779
**Correlation dimension D**_**2**_	<0.001*	0.584	0.879
**Vessel percentage area**	<0.001*	0.614	0.698
**Total number of junctions**	<0.001*	0.611	0.257
**Total vessel length**	<0.001*	0.617	0.881

* Values that are statistically significant

Liu et al. used Optical Coherence Tomography Angiography (OCTA) to compare the microvascular density between the left and right fellow eyes [32]. As in the present study, the authors noted a similar moderate correlation in microvascular density between the fellow eyes, but they also noted that the vascular density is higher in the right eye, which is the dominant eye in most of the people. Unlike Liu’s study we did not observe these differences in vascular density between the fellow eyes. This may be because the OCTA visualizes capillaries, while the digital fundus camera that we used shows mostly arteriolar and venuar portions of the microvascular network, and these differences may not be present in this segment of microvasculature. Also, our sample size was smaller than in Liu’s study.

### Demographic characteristics, social habits, and health status

Demographic characteristics, social habits, and health status for the TREND group and for the group of healthy older participants are shown in the Tables 4 and 5 respectively.

**Table 4:** Characteristics of the young, healthy participant population- independent variables associated with 72 fundus images in the TREND database.

Continuous	Mean±SD, (Min-Max)
**Age**	19.87±0.98, (19–23)
**Males by age**	19.75±0.97, (19–22)
**Females by age**	19.95±0.99, (19–23)
**BMI**	22.36±2.83, (18.81–30.68)
**Males by BMI**	24.17±2.97, (20.16–30.68)
**Females by BMI**	21.21±2.06, (18.81–26.42)
**Categorical**	**Percent by category**
**BMI**	17.67% overweight
	83.33% normal weight
**Sex**	38.89% male
	61.11% female
Alcohol use (up to 3 drinks* **3 times per week)**	29.17% yes
	70.83% no
**Smoking (up to 15 cigarettes/day)**	17.67% yes
	83.33% no
**Refraction error**	38.89% yes, (myopia n = 21, hypermetropia n = 7, -4.95D to +1.5D)
	61.11% no

* Equivalent of 30ml of hard liquor

**Table 5:** Characteristics of the healthy, older group- independent variables associated with 10 additional fundus images.

Continuous	Mean±SD, (Min-Max)
**Age**	57.7±2.45, (55–60)
**Males by age**	58.33±2.58, (55–60)
**Females by age**	56.75±2.22, (55–60)
**BMI**	27.77±4.31, (23.09, 38.06)
**Males by BMI**	26.74±2.92, (23.09,29.41)
**Females by BMI**	29.32±6.01, (24.38, 38.06)
**Categorical**	**Percent by category**
**BMI**	10% obese
	60% overweight
	30% normal weight
**Sex**	60% male
	40% female
Alcohol (up to 1 drink* **every day)**	50% yes
	50% no
**Smoking (up to 30 cigarettes/day)**	30% yes
	70% no (30% stopped smoking over 20 years ago, 40% never smoked)
**Refraction error**	60% yes, (myopia n = 0, hypermetropia n = 6, 0D to +4D)
	40% no

* Equivalent of 30ml of hard liquor

Two participants from the healthy and young group reported history of asthma, one history of bronchitis, and one history of celiac disease, but all of them denied having to take any medications for these conditions on regular basis. In addition, one participant reported that they had history of surgical correction of strabismus that resulted in partial correction of the problem (refraction error -4.25 on the left and -4.95 on the right).

All older participants denied history of diabetes, coronary artery disease, cerebrovascular disease, and neurological diseases including cognitive disorders. Two participants had a positive history for hypertension, but it was well controlled with medications. Other than refraction errors corrected with prescription glasses mentioned in the Table 5, other ocular history was negative for all older participants.

### The proof of the concept

The effect of age on retinal microvascular geometry by using fractal analysis parameters and blood vessel density parameters has been described in the past by other researchers [1, 20–22]. These studies showed that the microvascular complexity characterized by fractal dimension decreases with age. It is important to note that most of them did not find significant change of microvascular complexity associated with gender, BMI, use of alcohol, or with smoking.

**Fig 4:**
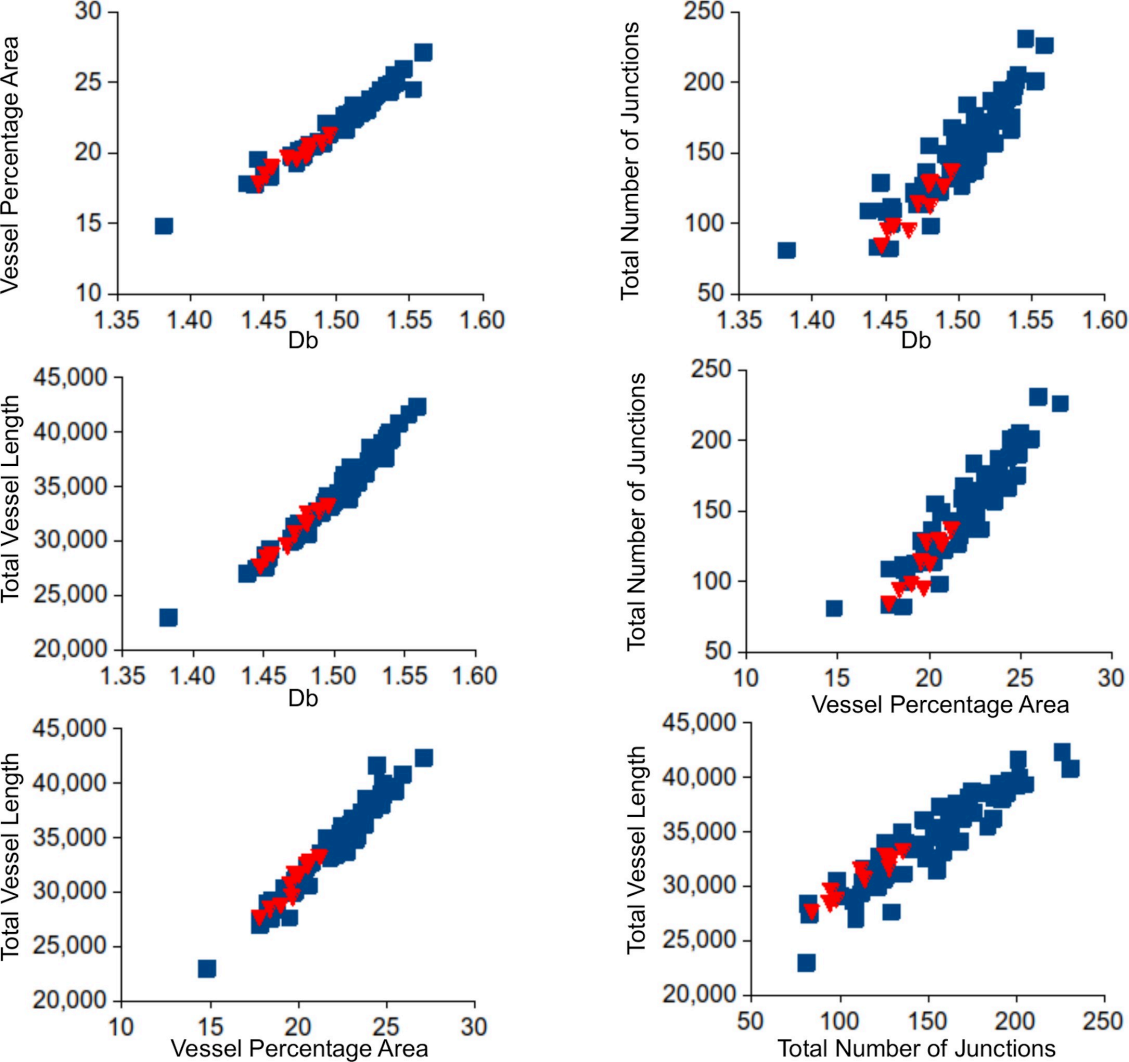
Two-dimensional scatter plots illustrating the clustering of the different retinal microvascular network patterns. The dependent variables that were examined are: box counting dimension D_b_ as a representative for fractal analysis results, vessel percentage area, total number of junctions, and total vessel length. All combinations of 2 different dependent variables are shown. The blue squares represent microvascular network patterns of the healthy young population (TREND), while the red triangles represent patterns of the healthy older population.

As a proof of concept, our present study shows that all measured variables except lacunarity dimension were significantly lower in older group of participants confirming that complexity of microvascular network decreases with the age (Table 6 and Fig 4), which is in agreement with findings of other researchers [1, 20–22]. In these studies, age was associated with microvascular complexity measured by fractal dimension independent of other variables.

**Table 6:** Comparison of microvascular parameters between old vs. young subjects and between subject with healthy BMI vs. overweight and obese BMI.

	**Old mean±SD** **(n = 72)**	**Young mean±SD** **(n = 10)**	**t-test p-value**
**Box-counting dimension Db**	1.472±0.016	1.498±0.032	0.013*
**Lacunarity dimension Λ**	0.451±0.036	0.441±0.035	0.393
**Capacity dimension D**_**0**_	1.525±0.016	1.552±0.027	0.003*
**Information dimension D**_**1**_	1.516±0.016	1.541±0.028	0.006*
**Correlation dimension D**_**2**_	1.51±0.015	1.534±0.028	0.010*
**Vessel percentage area**	19.668±1.046	21.833±2.343	0.005*
**Total number of junctions**	111.6±17.989	149.014±34.328	0.001*
**Total vessel length**	30629.448±1982.221	33997.651±3979.429	0.010*
	**Healthy BMI mean±SD (n = 63)**	**Overweight and obese BMI mean±SD (n = 19)**	**t-testp-value**
**Box-counting dimension Db**	1.499±0.030	1.482±0.035	0.037*
**Lacunarity dimension Λ**	0.440±0.034	0.451±0.039	0.212
**Capacity dimension D**_**0**_	1.551±0.026	1.539±0.030	0.081
**Information dimension D**_**1**_	1.541±0.027	1.528±0.029	0.077
**Correlation dimension D**_**2**_	1.534±0.027	1.522±0.029	0.096
**Vessel percentage area**	21.847±2.261	20.645±2.391	0.048*
**Total number of junctions**	147.698±34.208	133.789±36.156	0.129
**Total vessel length**	34030.513±3894.842	32116.513±3839.389	0.067

*Differences that are statistically significant

Gender, use of alcohol, and smoking did not have significant effect on microvascular patterns (data not shown) and this is in agreement with observations of others [1, 22]. Additionally, the use of tobacco and alcohol in young participants in our study may not be long enough to cause significant remodeling of retinal microvascular network.

The present study showed that based on the t-test analysis, the participants who have overweight and obese BMI have slightly lower box-counting dimension and vessel percentage area (Table 6). The 2-way ANOVA analysis showed there was no significant interaction between the age and BMI for any of the measured variables (Table 7). In comparison, studies by Cheung and Zhu did not find any association between age and BMI after they corrected for the presence of confounding factors. In addition to this, Van Creanendonk et al. found that a higher BMI is associated with increased fractal dimension of retinal microvasculature. Disagreements among the studies may be caused by the fact that populations examined in each study are different. For example, the mean age of the participants was 38 years in the Van Creanendonk’s study, while it was 51 in Zhu’s and 57 in Cheung’s study. At the same time, the Van Creanendonk’s study had participants with the mean BMI of 29, while in Zhu’s and in Cheung’s study it was 24 and 27 respectively. This is important information because higher BMI, especially in younger adult males in many cases may be the consequence of increased muscle mass, and not higher body fat content. While our study mean age and BMI for older participants are the most similar to Cheung’s study, the mean values of age and BMI for the younger group of participants do not match with any of these studies. Also, some of the risk factors like high BMI, smoking, alcohol use, were present in a relatively small proportion of participants in the present study, and the total number of participants was much smaller compared to the above mentioned studies, so the statistical tests with multivariate comparisons had low statistical power, and the multiple linear regression analysis could not be performed because the assumptions for this type of the test were not met.

**Table 7:** Results of 2-way ANOVA exploring the effects of age and BMI on microvascular parameters.

	Overall 2 way ANOVA p-value	Main effect of age p-value	Main effect of BMI p-value	Interaction Age X BMI p-value
**Box-counting dimension Db**	0.047*	0.110	0.178	0.499
**Lacunarity dimension Λ**	0.445	0.879	0.167	0.321
**Capacity dimension D**_**0**_	0.027*	0.019*	0.526	0.791
**Information dimension D**_**1**_	0.047*	0.037*	0.475	0.814
**Correlation dimension D**_**2**_	0.070	0.050	0.497	0.811
**Vessel percentage area**	0.031*	0.045*	0.285	0.586
**Total number of junctions**	0.011*	0.008*	0.489	0.434
**Total vessel length**	0.051	0.075	0.256	0.491

*Differences that are statistically significant

The causes for refractive error are heterogeneous and not all of them are related to aging [33]. However, it has been shown that people with high myopia who have refractive error greater -5D have decreased complexity of retinal microvascular network measured by fractal dimension [1, 34]. Therefore the information on refractive error was collected from each participant. None of the study participants had high myopia. In addition, there was no significant difference in microvascular patterns in eyes with refractive error compared to those without refractive error (data not shown).

### Database utility

#### Development of imaging biomarkers for certain systemic and eye- specific diseases

The utility of the TREND database is reflected in its potential for easy expansion of the number of images and its application in the development of new imaging biomarkers that could aid detection of various eye-specific, as well as systemic diseases. For example, in a recent study Poplin et al. used a large dataset of retinal images from 284,335 participants to develop a deep learning algorithm that was able to predict cardiovascular risk factors for an individual based on features that are present in their color digital retinal fundus photograph. As expected, the attention maps showed that this neural network model used areas of the image representing visible microvasculature, but often it also used other non-vascular features of the image to make the prediction [35]. The portability of the fundus camera and its ease of use will enable researchers to reach this large number of participants and not to be limited by the accessibility of the equipment. This research could improve our understanding of the pathophysiological mechanisms in development of certain diseases and improve monitoring of treatment outcomes.

We observed that the ease of use of the portable fundus camera could be hindered by the opacity of the clear eye media. Therefore, the use of this specific portable eye fundus camera could be the most beneficial for the early detection of the disease and disease-related complications in the younger and middle-aged population because the imaging success rate will be the highest. In addition, the impact of the treatment measures that are undertaken in early phases of the disease will be the greatest. It is important to emphasize that the use of a portable hand-held fundus camera for a general screening of the older population should not be excluded, but in the cases of unsuccessful imaging, those patients should be referred to the specialist for proper evaluation.

#### Development of biomarkers of accelerated aging

The normal aging process is characterized by changes in the biomechanical properties of the larger blood vessels. This process is accelerated by age-related chronic diseases like hypertension [36]. Similarly, at the microcirculation level, aging is associated with decreased complexity of retinal microvascular network, and this change is also noted in some age-related diseases like hypertension, cerebrovascular disease, and cognitive impairment [1, 7, 26]. Very often the changes of retinal microvascular geometry caused by age-related diseases could be considered as a process that normally occurs with aging, but at an accelerated speed. For example the study by DeBuc et al. showed that the microvascular complexity in the macular region of the retina is decreased to higher degree in individuals with cognitive impairment compared to healthy individuals of the same age [7].

In the recent years a new paradigm emerged that aging, age-related diseases, and geriatric syndromes such as fraility belong to the same spectrum of processes because they develop as a result of many common molecular mechanisms [37]. According to this theory, on one end of this spectrum are people who age at a healthy rate and live a long life, and on the opposite end are those who suffer from age-related diseases and geriatric syndromes, and therefore have accelerated aging. This phase of accelerated aging lasts for years due to functional redundancies that are naturally present in every living system. It precedes the overt clinical manifestation of the age-related disease and may be already present in middle aged people. This phase is non-specific because it shows that the risk for age-related disease is higher than normal, but it does not predict for which disease. Family medicine doctors, at the level of primary health care, are the ones who most frequently encounter patients in this early phase of the disease where symptoms are not fully developed [38]. Therefore, the detection of these individuals by a family medicine doctor during this critical time window would allow for the most effective interventions.

Several recent studies have demonstrated that specific patterns of DNA methylation detect accelerated aging, which manifested as an older “biological age” compared to the actual “chronological age” in patients with cardiovascular diseases, cancer, and neurodegeneration [39]. However, the use of these proposed markers of accelerated aging is still very expensive and time consuming. In this light, the use of retinal microvascular complexity could be explored as a potential imaging marker of accelerated aging. Fundus photography is noninvasive and inexpensive, and the application of computer-aided algorithms could significantly improve the speed of this method. The dataset provided in the current study provides an example where biological age is expected to be the same as chronological age because these participants are healthy.

#### Screening for classical complications of chronic systemic diseases in the primary health care setting

The use of a hand-held digital portable camera in primary practice as a part of screening for classical complications of chronic systemic diseases like diabetes and hypertension could be easy and simple, especially if there is support of an ophthalmologist who can review the images remotely. This is a convenient tool for the visualization of the eye fundus in individuals whose access to institutions of a higher level of healthcare is difficult because they are located in a remote area, or if they have decreased mobility. In this process, the healthcare worker’s familiarity with normal topological features of the eye fundus is of paramount importance.

In accordance with this, Vujosevic at al. reported very promising results of telematic screening implemented at the several locations in Italy as a part of efforts of this health care system to establish a national screening program for diabetic retinopathy [40]. Diabetic retinopathy is the leading cause for blindness. Therefore early detection and intervention is critical in preventing blindness [41]. This group of authors showed that the use of telemedicine improved the access to screening for the patients, the economic use of resources by health care institutions, and cost-effectiveness of the screening without compromising the care delivered to the patients. The authors also recommended that the number of fields captured by non-mydriatic camera should be increased to at least 2 or 3, in order to increase the sensitivity and specificity to the target levels necessary for an effective screening of diabetic retinopathy.

#### Computer-aided detection of retinal pathology

The TREND database could be used for computer-aided evaluation of image quality, the development of digital tools for semi-automatic detection of retinal pathology associated with various eye diseases, as well as for detection of retinal complications of some systemic diseases like hypertensive and diabetic retinopathy. Results of numerous research efforts in that regard have been already published. For example, a recent study in Spain has shown that semiautomatic measurement of the retinal microvasculature could be used to assess cardiovascular risk in hypertensive patients in retinal photographs that were collected using a portable fundus camera at a primary care physician’s office. This group of authors suggested that the use of semiautomatic measurement in the primary health care setting could eliminate the subjective component in the evaluation of images that was causing low inter- and intra-observer concordance in the detection of hypertensive retinopathy [42, 43].

Finally, since the TREND database consists of 72 raw color digital fundus images, and each image is associated with the manually segmented binarized image of microvascular network, it can be used as a ground truth for the development of software for the computer-based vascular network segmentation.

### Limitations

The TREND database has several limitations. It contains a relatively homogeneous data set collected only from the students enrolled at the Faculty of Medicine of the University of Montenegro, and it does not contain retinal images from young healthy people from various social and ethnic/racial backgrounds, which would more closely model the real-life situation. The demographic, health, and social habits in this paper is self-reported information without objective measurement. Finally, this database contains a relatively small number of digital color images. However, to our knowledge, there are not many publicly available databases of this size containing retinal fundus images captured by a hand-held digital non-mydriatic fundus camera where each raw image is associated with the manually segmented image of the microvascular network.

## Conclusion

The color digital fundus images from the TREND database could be used as a standard that defines normal retinal anatomy and microvascular network geometry in young healthy people as it is recorded with a digital hand-held portable non-mydriatic camera.

The use of this specific portable eye fundus camera could have the highest impact on the younger and middle-aged population because the imaging success rate will be highest, and the impact of the treatment measures that are undertaken in early phases of the disease will be the greatest.

In the future research, the database could be used 1) as a basis for the development of new imaging biomarkers of chronic systemic and eye-specific diseases [35]; 2) for the development of biomarkers of accelerated aging [7]; 3) for establishing telematic screening for complications of chronic systemic diseases in the primary health care setting [40]; and 4) for the improvement of the current standards for evaluation of quality of retinal images [9] as well as development of digital software tools for semiautomatic measurement of pathologic changes in the retina [42, 43].
